# BioVis Explorer: A visual guide for biological data visualization techniques

**DOI:** 10.1371/journal.pone.0187341

**Published:** 2017-11-01

**Authors:** Andreas Kerren, Kostiantyn Kucher, Yuan-Fang Li, Falk Schreiber

**Affiliations:** 1 Department of Computer Science, Linnaeus University, Växjö, Sweden; 2 Faculty of Information Technology, Monash University, Clayton, Australia; 3 Department of Computer and Information Science, University of Konstanz, Konstanz, Germany; Harbin Institute of Technology Shenzhen Graduate School, CHINA

## Abstract

Data visualization is of increasing importance in the Biosciences. During the past 15 years, a great number of novel methods and tools for the visualization of biological data have been developed and published in various journals and conference proceedings. As a consequence, keeping an overview of state-of-the-art visualization research has become increasingly challenging for both biology researchers and visualization researchers. To address this challenge, we have reviewed visualization research especially performed for the Biosciences and created an interactive web-based visualization tool, the BioVis Explorer. BioVis Explorer allows the exploration of published visualization methods in interactive and intuitive ways, including faceted browsing and associations with related methods. The tool is publicly available online and has been designed as community-based system which allows users to add their works easily.

## Introduction

Data visualization is increasingly important in the Biosciences as stated by O’Donoghue et al. [1]. A large number of new tools and methods for biological data visualization have been developed and published in various journals and conference proceedings in the last 15 years. As a consequence, keeping an overview of state-of-the-art visualization research is crucial for both biology researchers (who would like to use such techniques for their individual analysis of problems or presentation of data) and visualization researchers (who would like to develop novel techniques).

The challenge when learning more about biological data visualization is that the methods available differ in a number of aspects, such as types and properties of the data to be visualized, interaction methods, and the concrete tasks which should be solved by the method. Usually only visualization experts have a good overview of these methods. To address this challenge and provide a review of current visualization approaches, we have created an interactive web-based visualization tool, called BioVis Explorer, which is freely available at

http://biovis.lnu.se

**Fig 1:**
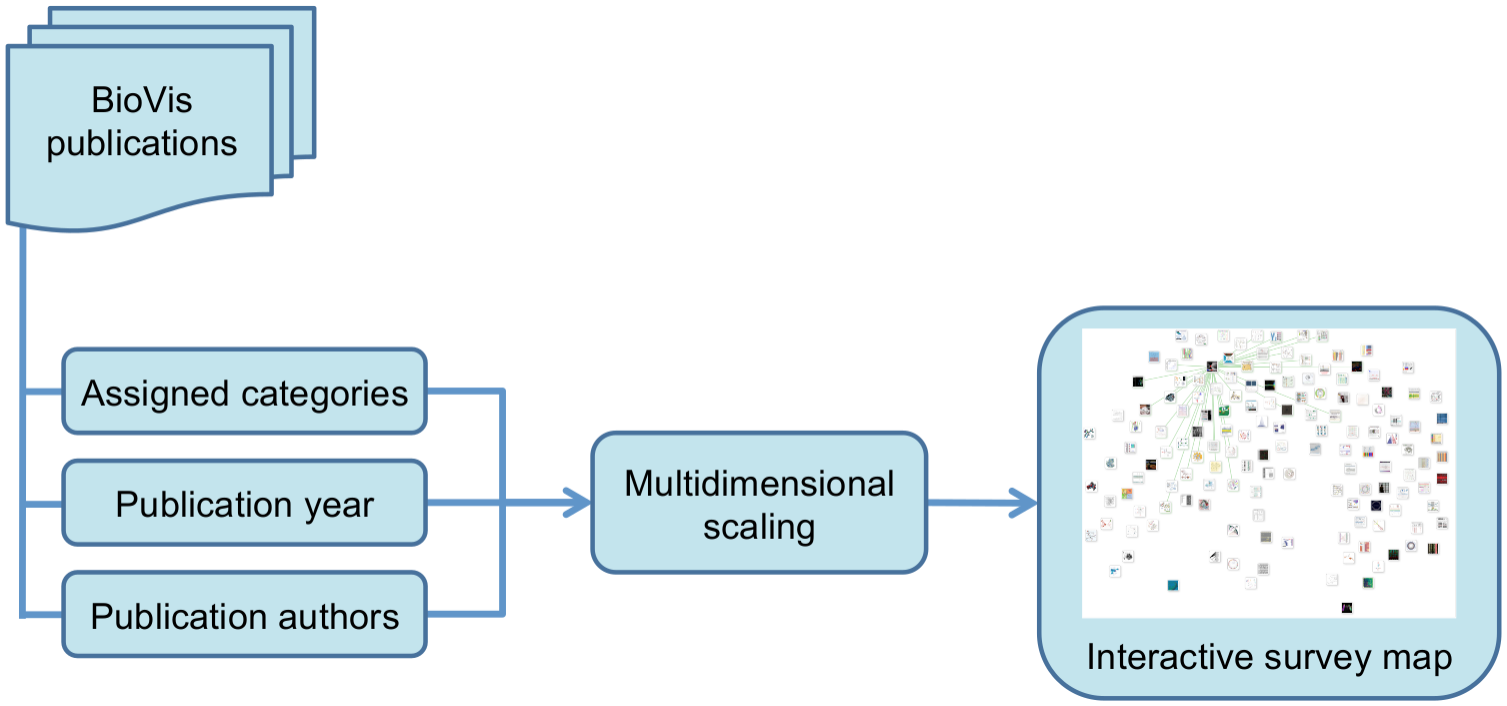
Overview of the approach used in BioVis explorer.

BioVis Explorer allows the investigation of visualization methods in intuitive ways based on filtering regarding, for example, types and properties of the data as well as associations with related visualization methods. The information about such categories is combined with the corresponding publication year and set of authors to provide an interactive map of visualization techniques, as illustrated in Fig 1. In this article—and consequently also in the developed interactive tool—we have focused on novel and dedicated visualization techniques for biological data sets proposed in publications from top-tier journals and conferences within a time range from 2000 to 2016. Therefore, we have not included general visualization methods [2], such as parallel coordinates plots or scatter plot matrices, as they were not developed originally for biological data sets. In order to be able to propose a manageable (i. e., relatively flat and narrow) taxonomy of various aspects of the data and visualization methods, we agreed on data usually occurring in systems biology [3], i. e., we are not considering other data sources that come from biomaterials or biomedicine, for example. A further reason for this decision was that systems biology heavily depends on efficient visualization methods that are able to support a better understanding of a biological system, ranging from visualizations for sequence analyses to visualization approaches for large and complex biological networks. In case there is a need for data sets coming from outside systems biology later on, we might extend our taxonomy accordingly. Moreover, we focus on visualization techniques and not on implemented systems which may quickly change over time. A system that is based on a specific technique presented in BioVis Explorer can develop over time and provide more data types or support more visualization tasks.

The remainder of this paper is organized as follows: first, we provide a brief overview of related work on interactive visual surveys/guides in general and of traditional surveys on visualization techniques for Bioinformatics in particular. Then, we highlight the overall design and important features of BioVis Explorer including a short description of the used methodology for collecting the data and implementation aspects. Finally, we present a use case scenario for showcasing the usefulness of our approach together with an expert review and information on utility/availability of the BioVis Explorer.

### Related (interactive) surveys

Regarding related work for the proposed interactive survey tool described in this manuscript, we distinguish between general interactive visual surveys (or guides) that focus on any research field and traditional surveys on Biovisualization techniques. To the best of our knowledge there is no interactive visual survey related to visualization techniques specifically developed for biological data. The list of visualization tools provided by the Biological Visualisation Network (BiVi, see http://bivi.co), for instance, focuses on systems and not on dedicated, peer-reviewed and published visualization techniques. Moreover, this list neither provides a fine-grained categorization as described in this work (only data type and more technical categories together with standard filter operations) nor supports more advanced analytical features such as the measurement of similarity between the techniques including its visualization.

#### Interactive visual surveys/guides

Over the last two decades, the field of Information Visualization has matured and it now provides a very large number of different techniques and tools. Due to the fast development of this and related fields such as Visual Analytics, traditional survey articles that give a more or less static overview of specific methods cannot be updated as frequently and as easily as the steady growth of visualization techniques demands. Therefore, recent surveys are either entirely web-based or—if they are published and printed—often accompanied by a survey website that is capable of including new research in a short period. An example for the former is a web-based survey on tree visualization methods [4]; whereas the latter approach is taken by the visual survey of time-dependent data visualization which is available in print [5]. Since the introduction of these initial interactive survey websites, many more have been launched, for example, for

- text visualization [6]
  (http://textvis.lnu.se),
- sentiment visualization [7]
  (http://sentimentvis.lnu.se),
- dynamic graph visualization [8]
  (http://dynamicgraphs.fbeck.com),
- set and set-typed data visualization [9]
  (http://setviz.net),
- performance visualization [10]
  (http://idav.ucdavis.edu/~ki/STAR/),
- multifaceted visualization [11]
  (http://multivis.net), or
- visualizing group structures in graphs [12]
  (http://groups-in-graphs.corinna-vehlow.com).

Note that the above mentioned TextVis Browser proposed by Kucher and Kerren [6] currently refers to 22 other web-based surveys in total. While not being a necessity for a survey paper, reviewers and readers of surveys in the visualization community frequently ask for making such an accompanying web resource available. There are also more generic approaches which may be instantiated with any scientific literature collection, such as the SurVis tool [13] (some of the above listed surveys are actually based on SurVis). Our proposed BioVis Explorer goes beyond these existing approaches by not just listing the literature in a specific order or providing standard filter features. We follow another layout strategy that spatially arranges the visual representations of the published articles according to similarity.

#### Surveys on biovisualization techniques

O’Donoghue et al. [1] greatly motivate the needs, trends, and challenges of using interactive visualization techniques in the Biosciences. Due to the sheer size, complexity, and heterogeneity of biological data, truly consolidated systems—in which several visualization techniques for a number of data types are integrated—are still rare. This fact is also reflected by existing surveys on Biovisualization that mostly focus on specific aspects in the data or on specific biological analysis tasks.

Many existing surveys on biological data visualization focus on biological networks in all of its variants. Kerren and Schreiber [14] present the state of the art in network visualization for the Biosciences together with a discussion of standards for the graphical representation of cellular networks and biological processes. Other examples are the somewhat older article of Pavlopoulos et al. [15] that also illustrates the functionality, limitations, and specific strengths of network visualization tools, the survey of Dinkla and Westenberg [16], and the presentation of graph and tree drawing algorithms for biological networks by Bachmeier et al. [17]. Other surveys focus on different areas such as visualizing time-dependent biological data [18], visualizing live cell imaging [19], visualizing microarray data [20], or visualizing spatial multivariate medical data with glyphs [21].

Neither of the aforementioned surveys provides a web-based interactive visual guide through the maze of various visualization techniques, nor encompasses the many techniques and similarity measurements as proposed in this work.

## BioVis explorer: Design and features

BioVis Explorer is an interactive web-based visualization tool that provides an electronic review of published visualization methods in interactive and intuitive ways, including faceted browsing and associations with related methods.

**Fig 2:**
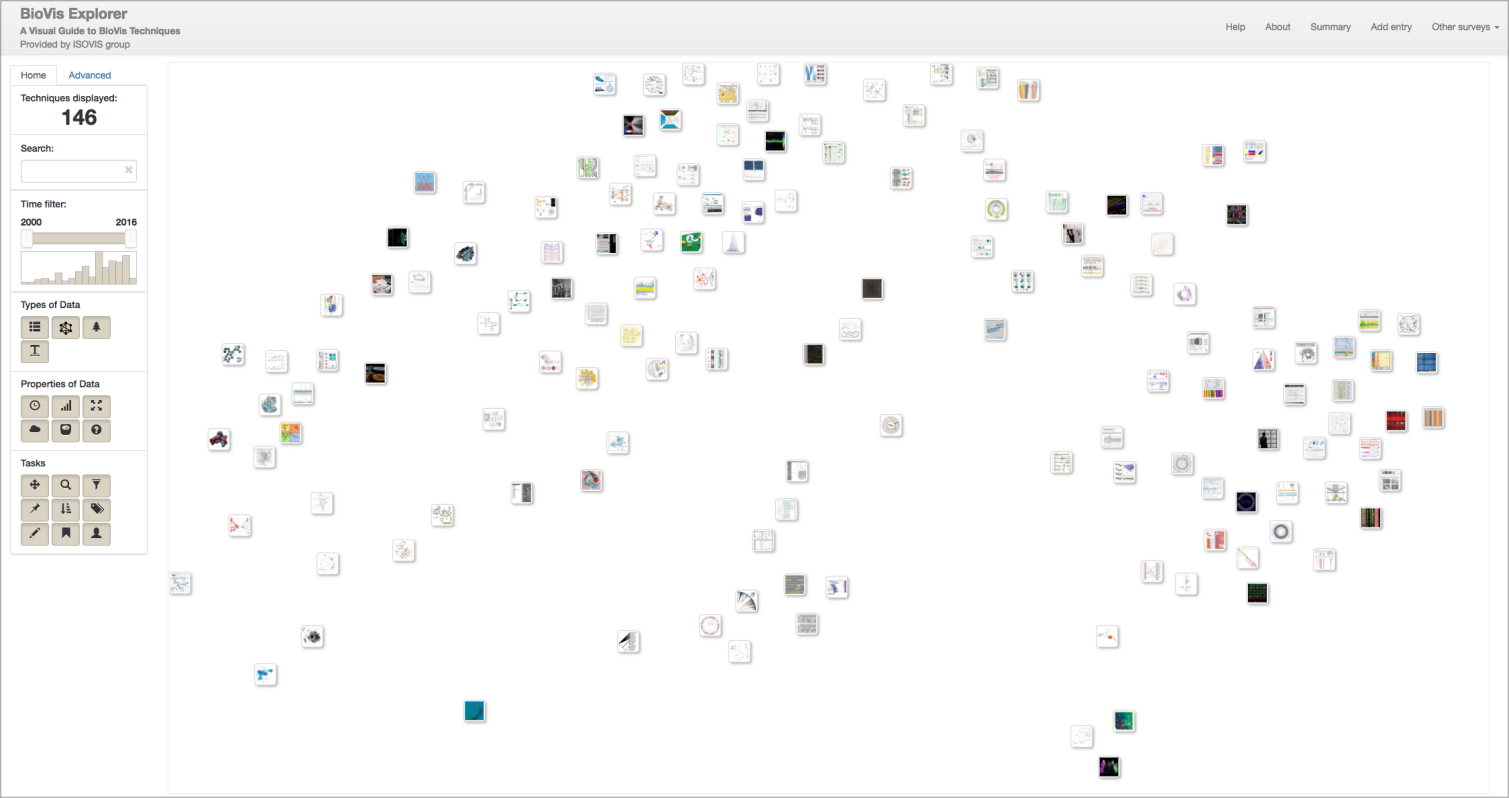
The web-based user interface of our interactive visual guide to BioVis techniques, called BioVis explorer. Each publication that presents a new BioVis technique is represented by a small rectangular thumbnail showing a cutout of the proposed visualization technique in the main view. The thumbnails are arranged in the 2D plane by using multidimensional scaling, i. e., similar techniques (according to a number of factors) are close to each other. By using the interaction panel on the left hand side, researchers can look for specific BioVis techniques and filter out entries with respect to a set of categories. The survey currently contains 146 categorized BioVis techniques published between 2000 and 2016.

Technically, BioVis Explorer is based on a galaxy metaphor [22] and provides a coherent view of BioVis publications in order to achieve a low initial learning curve (see Fig 2). The overall design of BioVis Explorer and its underlying architecture are inspired by our previous survey browser on text visualization [6]. Its interface comprises two main elements: a side panel and a main view. The side panel contains filters, statistics, and a temporal histogram chart that can be used for interactive exploration. The main view displays small thumbnails representing individual visualization techniques or papers, respectively. In contrast to many other online visual surveys discussed in the related work section, BioVis Explorer does not simply provide a thumbnail list ordered by a certain attribute, for instance, publication year. Instead, the elements are positioned according to the mutual dissimilarity of the corresponding entries, that is, thumbnails located close to each other correspond to techniques/publications with low distance (low dissimilarity). Thereby it is easy to get a quick overview of similar visualization methods. The 2D-layout of thumbnails is produced by dimensionality reduction—multidimensional scaling (MDS) [23]—for the matrix of distances between the techniques. The distance between a pair of techniques is computed as a weighted sum of the following factors: (i) difference in the publication year, (ii) difference in the assigned categories calculated by using the Jaccard index [24], and (iii) difference in the sets of authors for the corresponding pair of publications calculated by using the Jaccard index (the author names are normalized for this purpose to account for different spellings and abbreviations).

**Fig 3:**
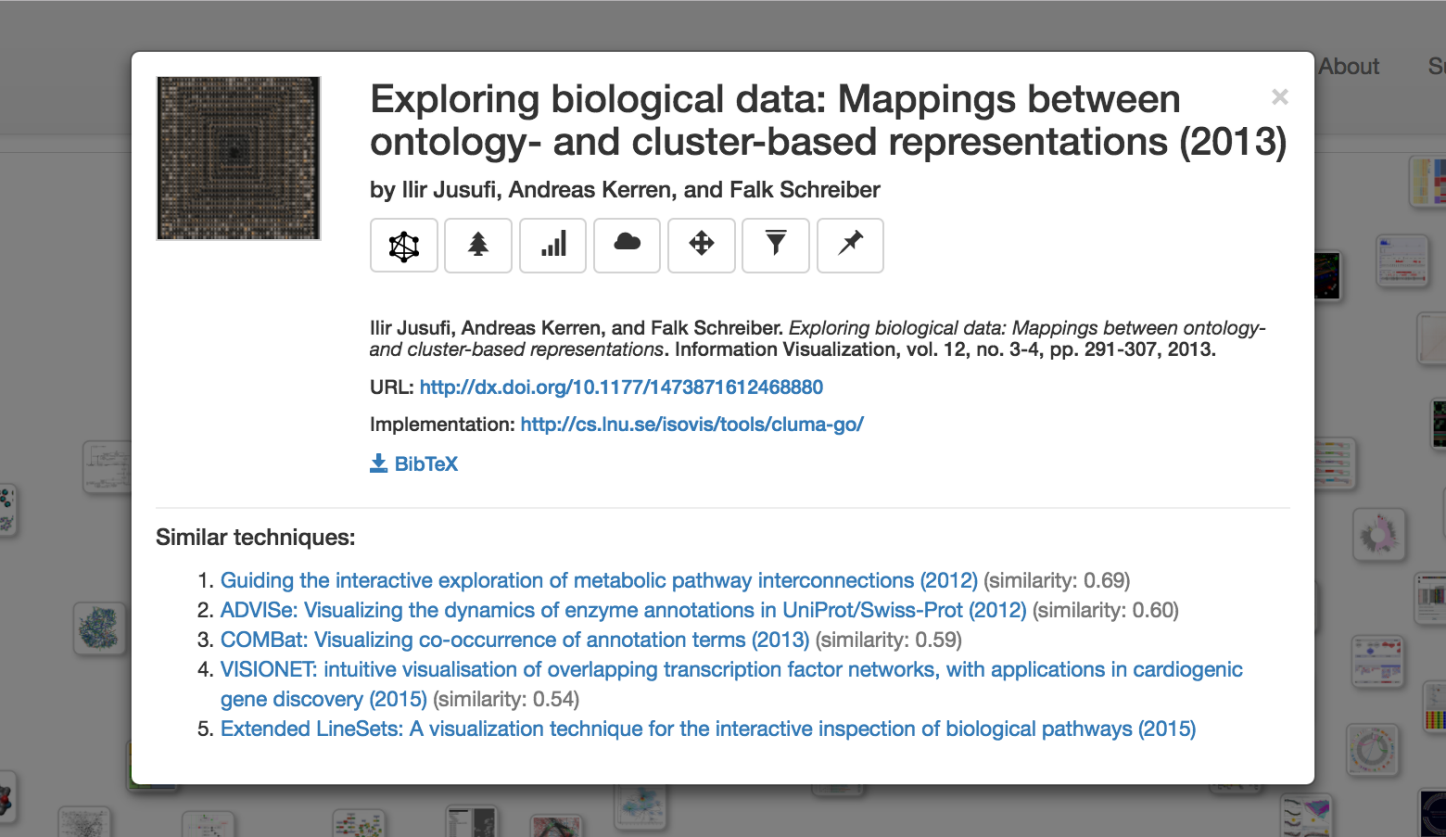
Detailed information of a publication title. After clicking on a thumbnail, the user can open a dialog with information on the technique title, corresponding types and properties of data, supported visualization and analysis tasks, corresponding publication, and a BiBTex file with bibliographical information. DOI-based publication URLs, implementation URLs, and PubMed IDs are also displayed if available. By clicking the title of a similar technique, the corresponding technique is displayed directly in the same dialog box.

**Fig 4:**
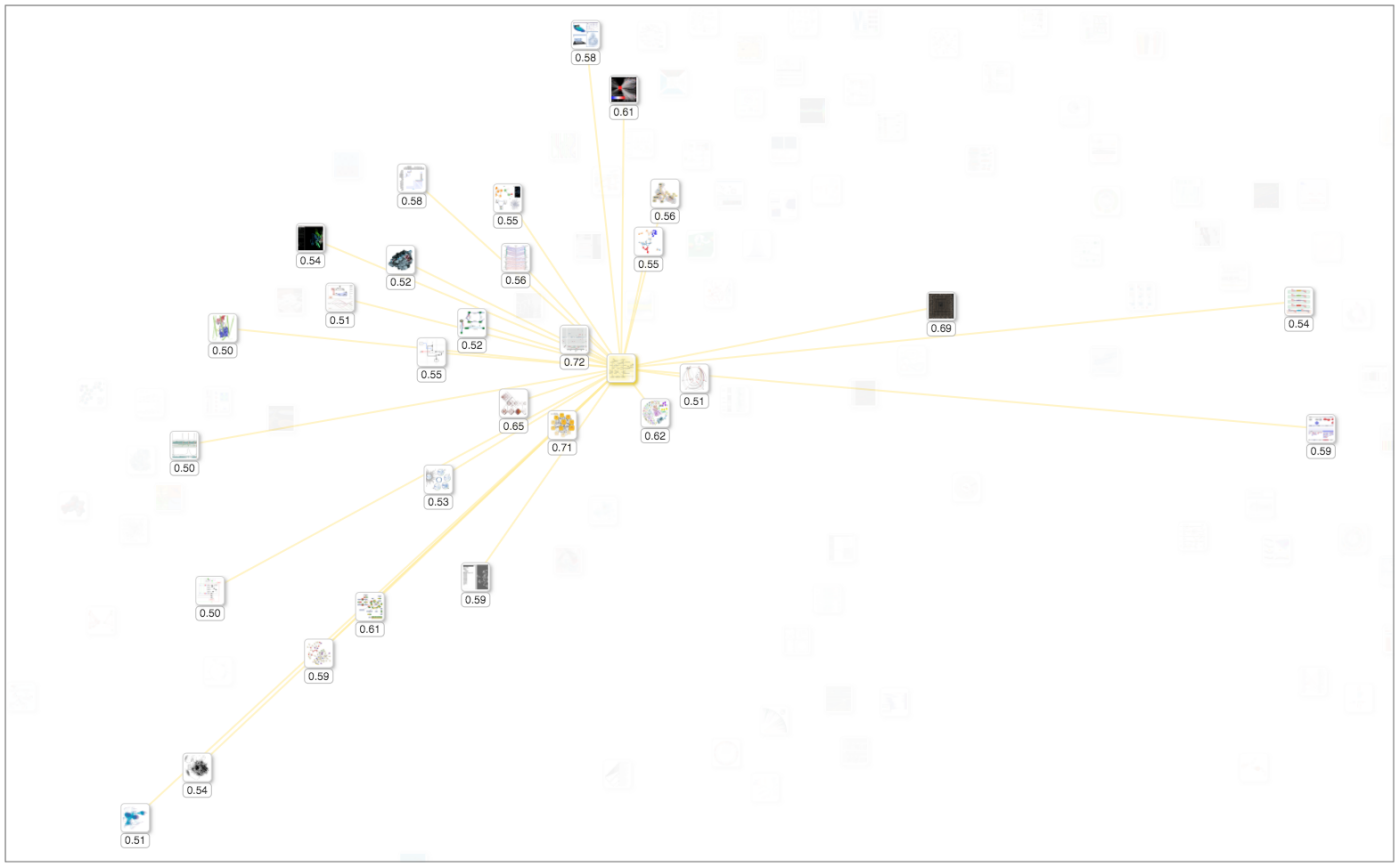
Individual comparison of a technique with similar ones. The selected technique thumbnail in the middle is highlighted with a yellow halo, and the connections to 29 similar techniques are shown. The small box below each thumbnail contains the actual similarity value. For instance, the thumbnail with value 0.72 is the most similar technique compared to the selected item.

BioVis Explorer supports several user interactions including zooming and panning. By clicking on a thumbnail, the user can open a dialog box with detailed information on the publication title, corresponding categories, and a file with bibliographical information, see Fig 3. This dialog also contains a list of similar techniques ordered by decreasing similarity; the similarity value being normalized in the range [0; 1] (here, 0 means minimum similarity / maximum distance, and 1 means maximum similarity / minimum distance). The user can also examine selected techniques and individually compare them with similar ones as shown in Fig 4. Hovering over a technique thumbnail blurs the thumbnails for dissimilar techniques and displays the links to similar techniques as well as the actual similarity value. In this way, the mental map of the thumbnail layout is not destroyed during the examination process. Right-clicking a technique thumbnail selects it and provides an effect similar to highlighting on hover, except for the fact that such selection is persistent until the same or another thumbnail is right-clicked.

**Fig 5:**
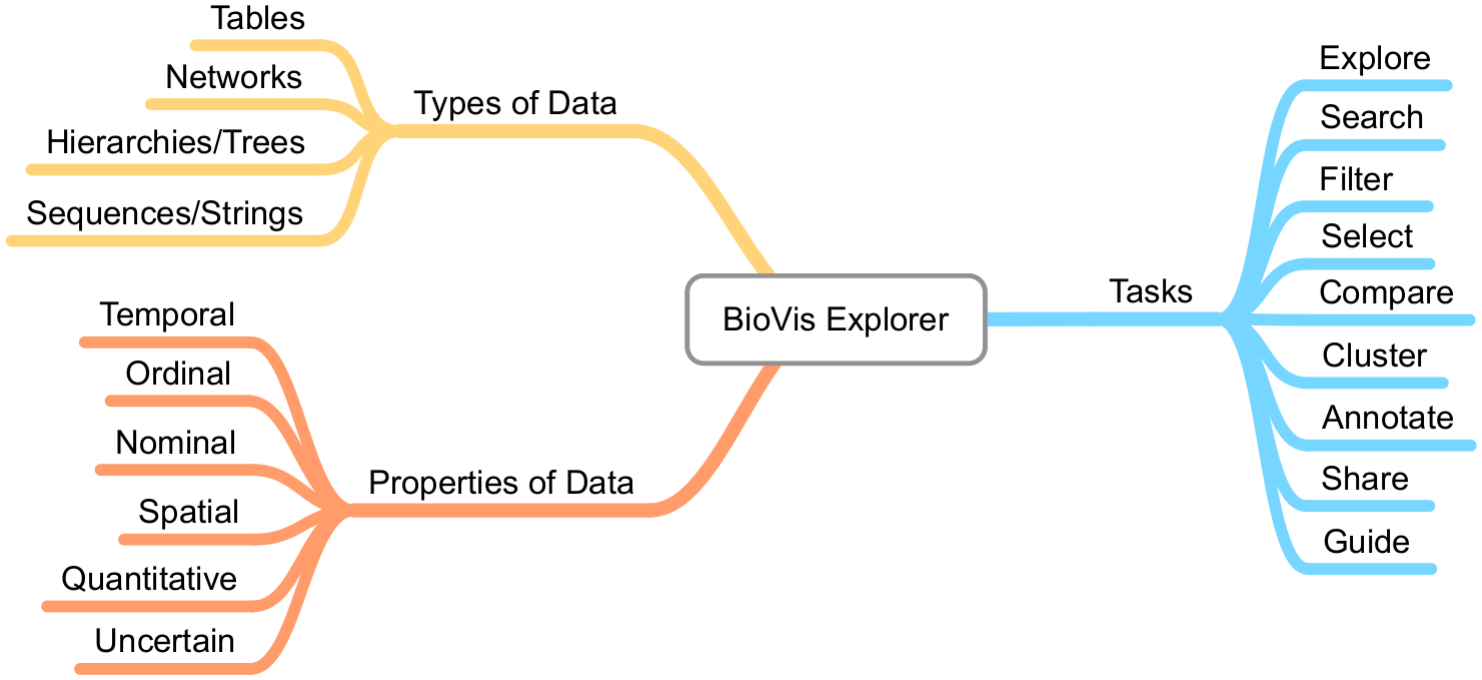
Taxonomy used by BioVis explorer.

The side panel shown on the left of Fig 2 provides several filtering techniques. Filtering can be performed according to keyword search, publication year, as well as a broad taxonomy. The taxonomy describes important facets of biological data visualization techniques, organized into three high-level categories: *biological data types* (i. e., the structure of the data when collected or stored), *biological data properties* (i. e., specific characteristics of the single data records that are typically not exclusive), and *visualization tasks* that a user might want to perform with the visualization. These categories are themselves divided again into subcategories as shown in Fig 5. We deliberately chose a relatively flat and simple taxonomy to make the usage of BioVis Explorer more intuitive and the visual display less cluttered. In the following, we showcase the categorization by means of two concrete example methods: *CluMa-GO* [25] which has been already shown in Fig 3 and *Circos* [26] which is the currently most cited technique in our data set according to Google Scholar and Web of Science. Note, that we do not show the results of our similarity analysis here, because they depend on the current parameter setting of BioVis Explorer that will be discussed further down in this paper.

**CluMa-GO** is a visualization approach for the combined display of an ontology and a hierarchical clustering of one data set (in the concrete use case of the paper: transcriptomics data). As the data consists of directed acyclic graphs and trees, “Networks / Graphs” and “Hierarchies / Trees” are suitable subcategories of biological data types together with the “Nominal” (genes) and “Ordinal” (hierarchically structured) data properties. Visualization tasks are in this case “Explore”, “Filter”, and “Select” due to the concrete interaction possibilities offered by the approach.
**Circos** has been developed to support the identification and analysis of similarities and differences arising from genome comparisons. The input data consists of genomic rearrangement data, hence “Networks / Graphs” and “Sequences / Strings” have been chosen as biological data types due to existing relationships between genomic intervals as well as the sequences themselves. Data properties are obvious in this case: “Nominal” (genes), “Ordinal” (ordered positions), but also “Quantitive” (probe values). Main visualization tasks are “Explore” and “Compare”.

**Fig 6:**
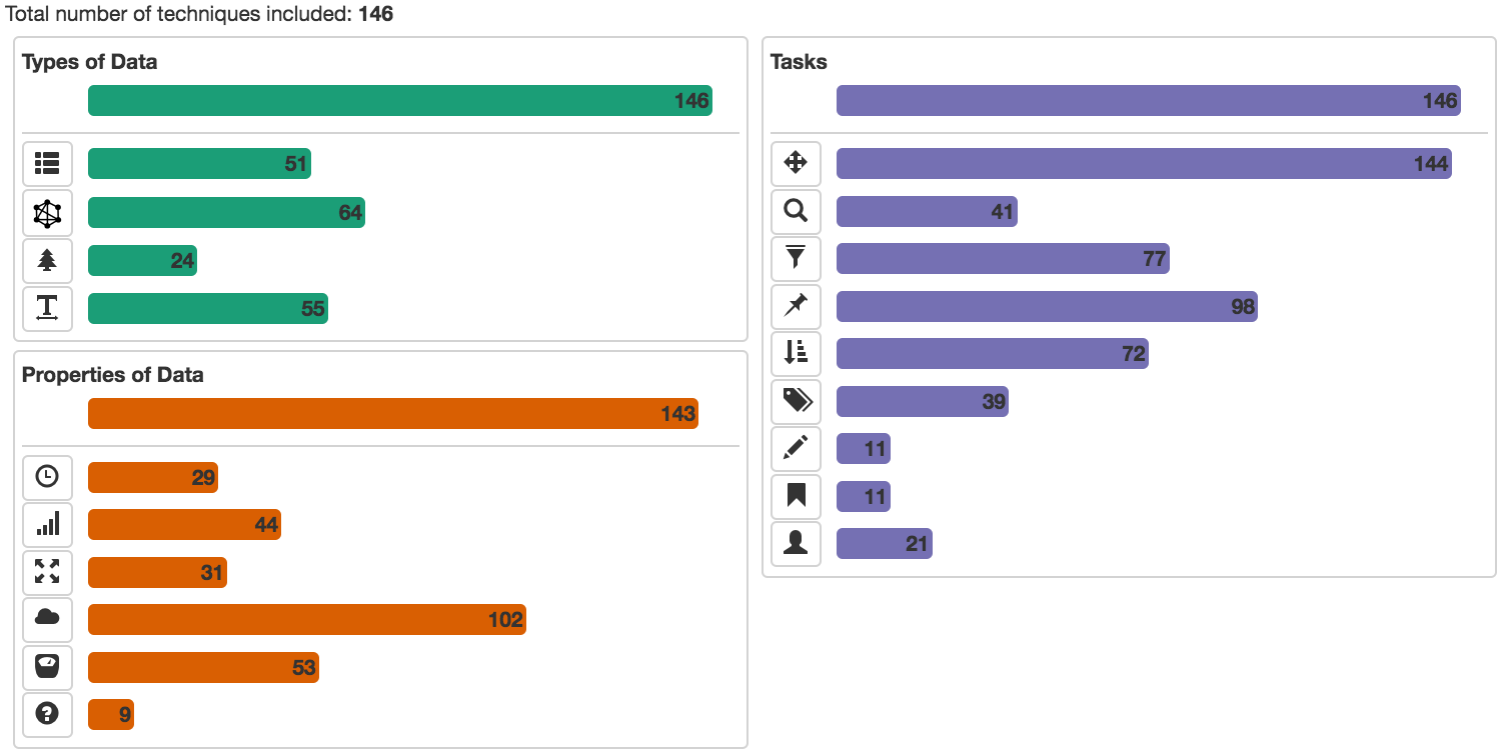
Category statistics for our current data set. The user can access the statistics for categories presented in Fig 5 by opening the “About” dialog.

An overview of all papers and their assigned categories can be shown on demand by clicking the “Summary” button (cf. S1 Table). The statistics for the categories are presented with bar charts in the “About” dialog as illustrated in Fig 6.

**Fig 7:**
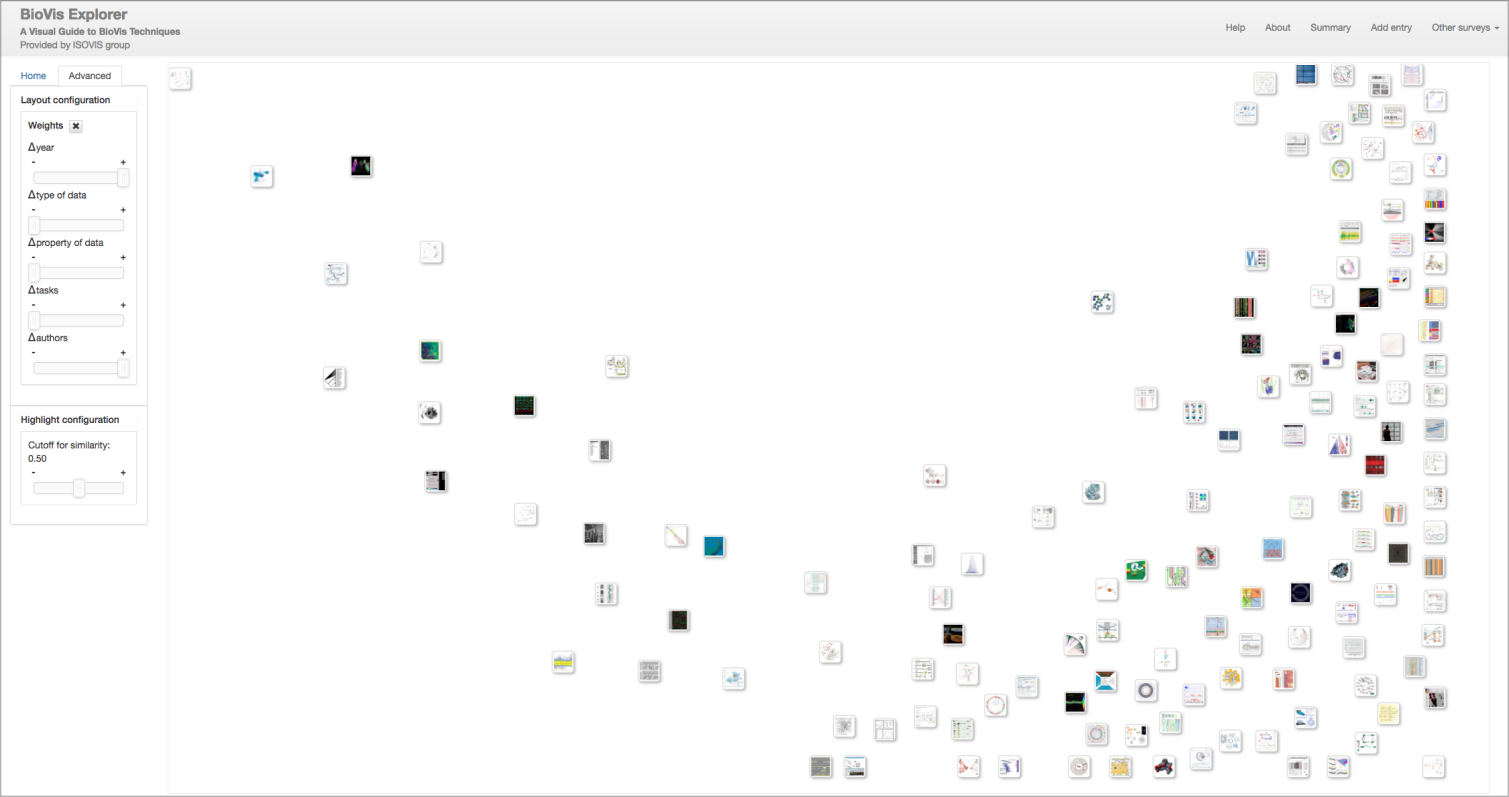
Giving distance factors another weight. Due to the currently chosen weights (all zero except for publication year and authors), the thumbnails were rearranged by the system.

The tool also provides more advanced settings for users interested in exploratory analysis. By adjusting the sliders for specific distance factors (see discussion above and Fig 7), users can change the weights of the factors when computing their dissimilarity, for instance, to reduce the importance of one or more factors, such as co-authorship. Setting a value to 0 will essentially exclude the factor from any computation. In consequence, the arrangement of thumbnails will be recomputed using the altered combination of factors. In the current screenshot of Fig 7, the weights for year and authors have been maximized whereas all others were minimized. The thumbnails have thus been rearranged so that older and less connected papers (with respect to common authors) appear on the left hand side, and newer papers with higher author connections appear on the right hand side. Again, thumbnails are close to each other if both factors are similar at the same time. It is also possible to adjust the value for the similarity cutoff/threshold to control which techniques will be considered similar or dissimilar during interactive exploration. Our supplementary video S1 Video demonstrates this behavior in action.

### Data collection process and tool implementation

BioVis Explorer is aimed to be a community-based system that offers the biovisualization community the possibility to enter and classify new work as described later in this section. As a starting point, a subset of existing publications that describe new methods for biovisualizations have been selected and already classified by three of the authors of this paper, all with several years up to decades of experiences in the area of visualizing biological data. Before the search process started, we agreed on an initial set of publication venues (top-tier journals/conferences; no workshops) and paper types (only full/short papers; no posters). In addition, we mainly considered publications that are accessible by most university libraries. Thus, for instance, we excluded for the time being less visible conferences, such as the EG/VGTC Conference on Visualization (EuroVis), because the Eurographics Digital Library or the affiliated journal Computer Graphics Forum are not always accessible by biologists/bioinformaticians in contrast to IEEE which has contracts with most libraries according to our experiences. Based on these rules, we searched for suitable publications from online databases provided by the corresponding paper/article publishers as listed in the following:

- Bioinformatics
  (http://bioinformatics.oxfordjournals.org)—currently, 41 techniques included in our survey;
- BioVis Conferences
  (http://biovis.net)—42 techniques;
- BMC Bioinformatics
  (http://bmcbioinformatics.biomedcentral.com/)—23 techniques;
- IEEE Conference on Visual Analytics Science and Technology (VAST)
  (http://ieeevis.org)—3 techniques;
- IEEE Information Visualization Conference (InfoVis)
  (http://ieeevis.org)—3 techniques;
- IEEE Pacific Visualization Symposium (PacificVis)
  (http://pvis.org)—2 techniques;
- IEEE Transactions on Visualization & Computer Graphics
  (https://www.computer.org/csdl/trans/tg/)—20 techniques; and
- Information Visualization Journal
  (http://journals.sagepub.com/home/ivi)—10 techniques.

It should be noted, that in single cases, we added publications that do not fall into these publication outlets, but have very high visibility in the field, e.g., the paper on Circos by Krzywinski et al. [26]. The process of assigning categories to papers can be summarized as follows: first, we designed and agreed on the taxonomy described in the previous section and shown in Fig 5. After that, we discussed the individual categories by means of sample papers to get a common understanding of what exactly is meant by the individual categories in order to preferably ensure a similar categorization process for each person who classified the publications. The next step was to select a time range between 2000 to 2016 and to partition the search space by assigning outlets to the individual expert for the classification process. Each expert then searched for suitable publications proposing novel visualization techniques for biological data and classified them according to our taxonomy, i. e., relevant papers were studied and categories were assigned based on manual evaluation of paper by three authors of this paper. Unclear cases were discussed together via email and/or virtual meetings. All results were collected in a shared spreadsheet to that everybody could follow the process and classification results of the others. This spreadsheet was finally used as input data set for our tool.

BioVis Explorer was implemented as a single-page web application with only static files hosted on the server, including the data set stored in the JSON format.

**Fig 8:**
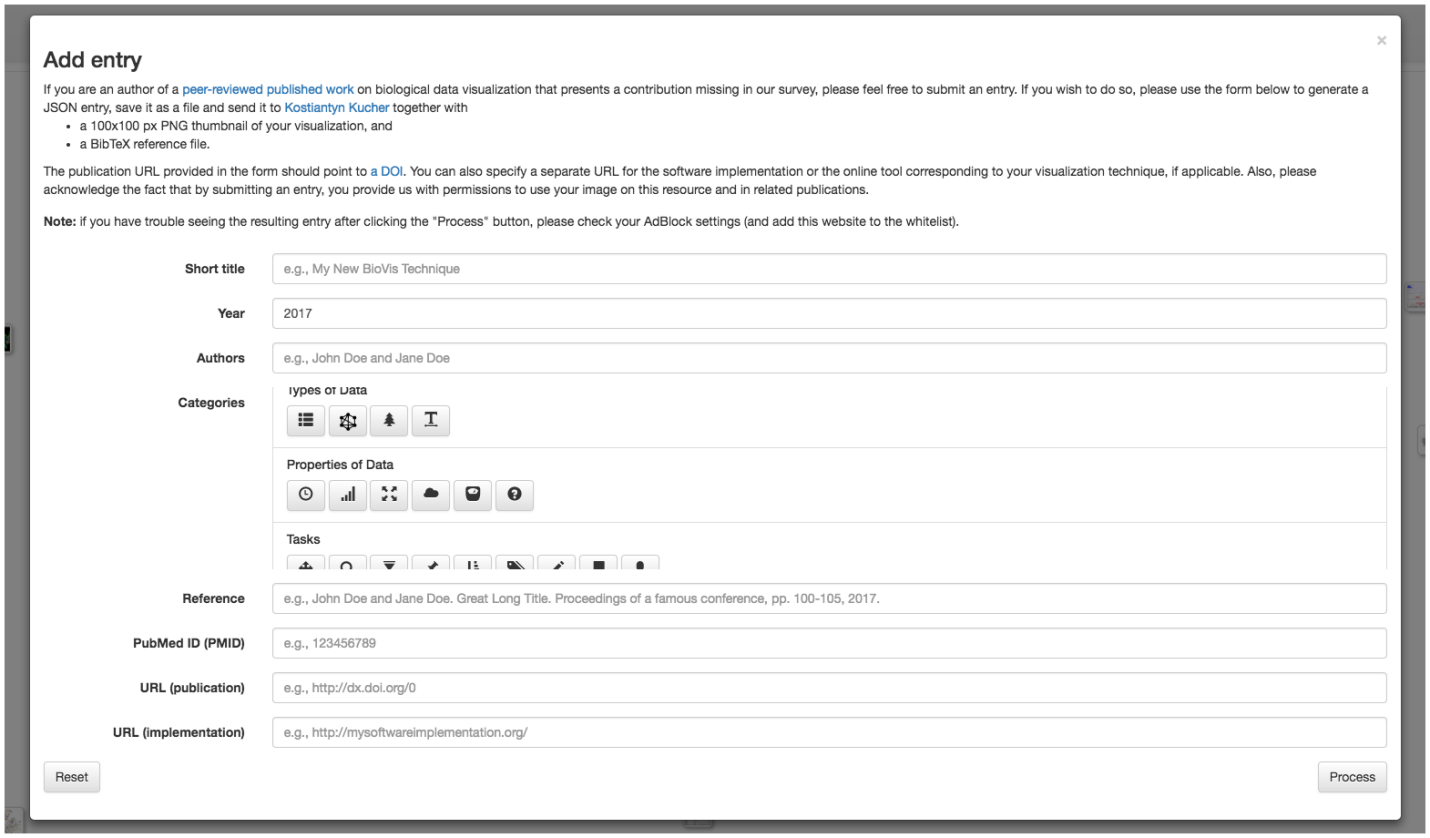
The dialog for submitting new visualization technique entries to our survey. The user can assign the corresponding categories and specify attributes such as technique title, authors and publication year. The optional fields include PubMed ID, publication URL and software implementation URL.

All interactive features were implemented with JavaScript and D3 (https://d3js.org) on the client side. In order to support the user submissions to the survey, BioVis Explorer includes a dialog shown in Fig 8, which is opened by clicking the “Add entry” button as can be seen at top right of Fig 2. This dialog contains fields similar to the ones displayed in the details dialog in Fig 3, including buttons for assigning categories from our taxonomy. By clicking the “Process” button, the user can generate a new JSON entry and send it to us via email alongside a corresponding thumbnail image and a BibTeX entry. All newly proposed entries are double-checked before making them publicly accessible in BioVis Explorer. This way, we can include new biological data visualization techniques suggested by the community, which are described in peer-reviewed publications from a wide range of outlets beyond our initial list described above.

## Discussion

In order to validate the overall visualization design and usability, we present a typical use case as well as an expert mini-review in this section. It is followed by a discussion on utility and availability of BioVis Explorer.

**Use case** A typical use case would consist of a researcher identifying the type and property of data and the research question or task of interest. Based on this information, the corresponding visualization methods will be presented and can be explored by the user. For example, a user is interested in finding all methods which are suited to compare and visualize networks which contain temporal (e. g., time series) data. First, he/she would filter out every data type which is not a network (or relational data) by deselecting all data types apart from networks/graphs. Next, the researcher would remove all methods which do not consider temporal data by deselecting all data properties other than temporal. Finally, he/she would remove (deselect) all tasks apart from compare. This currently results in ten different visualization methods, which could be explored by hovering over and clicking onto the small rectangular thumbnails; or the user could further restrict the presented visualization methods to the most recent ones by using the time slider. Finally, the user can follow the web-link to the publication which may offer a tool for the visualization method or a detailed description of the algorithm to visualize this specific combination of data type, data property and task.

**Expert review** Domain expert reviews are one option to get an impression of the usability of a visual interface and to identify potential problems without the need to perform a full-fledged user study [27]. To obtain initial feedback for evaluating and improving BioVis Explorer, we asked three biologists (on postdoc and PhD student level) who use data visualization regularly in their work tasks to provide feedback about the tool’s usability and usefulness. After a short (ca. 5 minutes) introduction, each subject was asked to work with the tool to explore biovisualization methods which may be relevant to his/her work and to provide answers to a set of given questions. There were three question categories: (i) questions regarding general information such as typical input data used, typical tasks to solve, and currently used visualization approaches, (ii) a mini-review of BioVis Explorer in the categories visual quality, ease of interaction, cost effectiveness, and usefulness to obtain information (each rated either “positively”, “neither nor”, or “negatively”), and (iii) free text comments regarding the tool.

The overall opinion of the domain experts was positive regarding visual quality, ease of interaction, and usefulness to obtain information, including comments such as “It is very neat to get an overview on similar tools with the small pictures of the visualization” or “It makes the search for a visualization technique very fast”. Of course, we also received more critical feedback, such as that it was not always clear what is meant with the categories (e. g., task: annotate), minor problems with standard interactions like which areas can be scrolled or not, or what exactly can be searched with the search function (i. e., words in titles or abstracts, keywords, etc.). A few of the problems have been already solved in the current version (e. g., “The description of the methods vanish too fast” was quickly solved by increasing the time this information is presented). For almost all perceived problems, it should suffice to provide a better and more meaningful online help. As the above mentioned search is restricted to titles, a next version of BioVis Explorer could be extended by considering keywords explicitly given in the papers. For doing that, we have to extend the collected data set however.

**Utility** The ISOVIS group at Linnaeus University will be responsible for the maintenance and long-term utility of BioVis Explorer. As the tool is a web-based application, any needed modification can be easily done on our web server without the need to disseminate any kind pf update information or files. The application is light-weight and can run on any modern web server. As the current hosting is located on a virtual server maintained by the university, we can guarantee its stability in the long run.

Future growth of the displayed data set is supported by the tool itself as new entries are based on the user’s/author’s self submissions as shown in Fig 8. This way, the system will also display work from other venues, such as from the EuroVis conference series, or other journals. Through small modifications in the Javascript code, we are also able to extend the taxonomy if there is a need for that.

**Availability** Actually, by following the URL of BioVis Explorer, all scripting files including the complete (current) data set are loaded into the web browser of the user and can be locally stored and re-used. If wished, we also can send the source files via email to interested people. Alternatively, we could provide a link to an automatically generated ZIP-archive for downloading the latest version of the tool and data files. Please note that the tool depends on the open source D3.js JavaScript library (https://d3js.org).

## Conclusion

BioVis Explorer helps researchers to investigate which visualization methods may be useful for a specific type of data or a specific analysis task within systems biology. Given the initial expert review and our overall positive experiences with this web-based system, we are confident that it will positively influence the increasingly data-driven analyses and subsequent findings within the different areas of systems biology. In addition, our work might also serve as promoter for the development of more efficient computational analysis tools for various analytical tasks, such as sequence analysis or the analysis of system dynamics, which use the visualizations discovered via BioVis Explorer on their part.

BioVis Explorer is freely available, accessible online without any user registration, and updates can be suggested by generating new entries with meta data (year, classification, etc.). In addition, the underlying source code is also freely available on request.

BioVis Explorer can be extended in a number of different directions. Firstly, ranking of visualization techniques, according to some bibliometrics, can be incorporated to provide users another way of exploring these techniques. Secondly, as we discussed earlier, the taxonomy can be updated to be more granular. This process can be manual or semi-automated through taxonomy learning. Thirdly, automated technique discovery and addition can be investigated. By crawling relevant publication venues and making use of information retrieval, natural language processing and machine learning techniques, the system could learn to self-update, hence reducing the need for manual addition.

## Supporting information

S1 Table Summary over all classified survey entries. To provide the user with an overview of all entry classifications, BioVis Explorer shows a tabular consisting of paper title (ordered according to publication year) and corresponding classifications in the shape of a matrix display. For seeing this, the user has to click the “Summary” button in the right upper corner of BioVis Explorer. The corresponding contents as of June 1, 2017 are provided in this table.
(PDF)

S1 Video Supplemental video demonstrates the usage of the visualization tool. (MP4)
