# Supplementary material for: BioVis Explorer: A visual guide for biological data visualization techniques
Source: PLoS One. 2017 Nov 1;12(11):e0187341. doi: 10.1371/journal.pone.0187341 (PMC5665558; doi:10.1371/journal.pone.0187341)
Supplement: S1 Table — To provide the user with an overview of all entry classifications, BioVis Explorer shows a tabular consisting of paper title (ordered according to publication year) and corresponding classifications in the shape of a matrix display. For seeing this, the user has to click the “Summary” button in the right upper corner of BioVis Explorer. The corresponding contents as of June 1, 2017 are provided in this table. (PDF) [file pone.0187341.s001.pdf]

BioVis Explorer (http://biovis.lnu.se) — survey summary (as of June 1, 2017)

Technique																			
Visualizing large hierarchical clusters in hyperbolic space (2000)		■		■				■			■	■		■					
BioLayout - an automatic graph layout algorithm for similarity visualization (2001)		■						■			■								
Visualizing Biosequence data using Texture Mapping (2002)				■				■			■				■				
Patika: an integrated visual environment for collaborative construction and analysis of cellular pathways (2002)		■					■	■			■	■							
Case Study: Visualizing Sets of Evolutionary Trees (2002)			■		■			■			■			■	■	■			
Global Visualization and Alignments of Whole Bacterial Genomes (2003)				■				■			■				■				
A fast layout algorithm for protein interaction networks (2003)		■						■			■								
Coordinated Graph and Scatter-Plot Views for the Visual Exploration of Microarray Time-Series Data (2003)	■				■				■		■	■	■	■					
GeneVis: Simulation and Visualization of Genetic Networks (2003)		■			■			■			■				■				
An Interactive Visualisation for Investigating DNA Sequence Information (2004)				■		■		■			■		■	■					■
Visualizations for taxonomic and phylogenetic trees (2004)				■				■			■	■							
Integration of metabolic networks and gene expression in virtual reality (2005)	■	■						■	■		■		■			■			
A system for visualizing and analyzing near-optimal protein sequence alignments (2005)				■		■		■			■		■	■					
Exploratory visualization of array-based comparative genomic hybridization (2005)	■					■	■	■	■		■	■							
ProViz: protein interaction visualization and exploration (2005)		■						■			■			■	■				
VitaPad: visualization tools for the analysis of pathway data (2005)		■						■			■			■					
Extending taxonomic visualisation to incorporate synonymy and structural markers (2005)			■					■			■			■	■				
A Framework for Visualization of Microarray Data and Integrated Meta Information (2005)	■					■		■	■		■								■
Animated interval scatter-plot views for the exploratory analysis of large-scale microarray time-course data (2005)	■				■				■		■		■	■					
Paloverde: an OpenGL 3D phylogeny browser (2006)			■								■						■		
springScape: visualisation of microarray and contextual bioinformatic data using spring embedding and an ‘information landscape’ (2006)	■		■		■			■	■		■								
TreeQ-VISTA: an interactive tree visualization tool with functional annotation query capabilities (2007)			■					■			■	■	■	■		■			
Exploring Multiple Trees through DAG Representations (2007)			■					■			■	■		■					
GOlorize: a Cytoscape plug-in for network visualization with Gene Ontology-based layout and coloring (2007)		■						■			■								
Cerebral: a Cytoscape plugin for layout of and interaction with biological networks using subcellular localization annotation (2007)		■					■	■			■					■			
QTLNetwork: mapping and visualizing genetic architecture of complex traits in experimental populations (2008)	■			■		■		■		■	■	■							
Presenting and exploring biological pathways with PathVisio (2008)		■					■	■			■								
Dynamic Visualization of Coexpression in Systems Genetics Data (2008)		■						■			■	■	■	■		■			
TreeMos: a high-throughput phylogenomic approach to find and visualize phylogenetic mosaicism (2008)				■				■			■			■		■			
VistaClara: an expression browser plug-in for Cytoscape (2008)	■				■		■		■		■	■	■	■	■				
NetworkBLAST: comparative analysis of protein networks (2008)		■													■				
PhyloWidget: web-based visualizations for the tree of life (2008)			■					■			■			■					
Interactive visualization software for exploring phylogenetic trees and clades (2008)			■					■		■	■		■	■	■	■			■
Cerebral: Visualizing Multiple Experimental Conditions on a Graph with Biological Context (2008)	■	■			■			■	■		■		■	■	■				
Caleydo: Connecting Pathways and Gene Expression (2009)	■	■						■	■		■	■	■	■		■			
Treevolution: visual analysis of phylogenetic trees (2009)			■					■			■					■			
Using Projection and 2D Plots to Visually Reveal Genetic Mechanisms of Complex Human Disorders (2009)	■								■		■		■						
ABySS-Explorer: Visualizing Genome Sequence Assemblies (2009)				■				■			■								
MizBee: A Multiscale Synteny Browser (2009)				■	■			■			■		■	■	■				
AssociationViewer: a scalable and integrated software tool for visualization of large-scale variation data in genomic context (2009)				■				■			■	■	■	■					
PhyloDet: a scalable visualization tool for mapping multiple traits to large evolutionary trees (2009)			■			■		■			■								
Circos: An information aesthetic for comparative genomics (2009)		■		■		■		■	■		■								
MassVis: Visual Analysis of Protein Complexes Using Mass Spectrometry (2009)	■								■		■	■	■	■	■				
GeneShelf: A Web-based Visual Interface for Large Gene Expression Time-Series Data Repositories (2009)	■	■			■			■	■	■	■		■	■	■				
BioCichlid: central dogma-based 3D visualization system of time-course microarray data on a hierarchical biological network (2009)	■	■			■						■								
A novel method for large tree visualization (2009)			■					■			■	■				■			
SpRay: A Visual Analytics Approach for Gene Expression Data (2009)	■								■		■		■	■					
GeneTerrain: Visual exploration of differential gene expression profiles organized in native biomolecular interaction networks (2010)	■	■						■	■		■			■					

Technique																			
Interactive microbial genome visualization with GView (2010)				■		■		■			■			■					
Gremlin: An Interactive Visualization Model for Analyzing Genomic Rearrangements (2010)				■		■		■		■	■			■	■				
MulteeSum: A Tool for Comparative Spatial and Temporal Gene Expression Data (2010)	■			■	■		■	■	■		■		■	■	■				
Visual Integration of Quantitative Proteomic Data, Pathways, and Protein Interactions (2010)	■	■						■	■		■	■	■	■		■			
iHAT: interactive Hierarchical Aggregation Table (2011)			■	■		■		■	■		■		■		■				
Visualization of Anisotropic Contact Potentials within Protein Structures (2011)			■				■				■				■	■			
MGV: a generic graph viewer for comparative omics data (2011)	■	■			■		■	■	■	■	■		■	■	■	■			
Automatic generation of protein structure cartoons with Pro-origami (2011)				■		■					■								
Cytoscape 2.8: new features for data integration and network visualization (2011)		■			■	■			■		■	■	■	■					■
RuleBender: Integrated Visualization for Biochemical Rule-Based Modeling (2011)		■					■				■		■	■	■				
Visual Analysis of Next-Generation Sequencing Data to Detect Overlapping Genes in Bacterial Genomes (2011)				■		■					■		■	■					■
Creating views on integrated multidomain data (2011)	■	■			■		■	■	■		■	■	■	■					■
Interactive, multiscale navigation of large and complicated biological networks (2011)		■						■			■		■	■		■			
MDMap: A System for Data-Driven Layout and Exploration of Molecular Dynamics Simulations (2011)		■			■						■	■	■			■			■
Evaluating the VIPER Pedigree Visualisation: Detecting Inheritance Inconsistencies in Genotyped Pedigrees (2011)	■		■	■		■			■		■		■	■	■				
EVEVis: A Multi-Scale Visualization System for Dense Evolutionary Data (2011)	■	■	■		■		■		■		■		■	■		■			
Metrics for Comparing Explicit Representations of Interconnected Biological Networks (2011)		■					■								■				
A Visual Analysis System for Metabolomics Data (2011)	■						■				■		■	■	■				■
GenPlay, a multipurpose genome analyzer and browser (2011)				■		■		■	■		■	■	■		■	■			
Parallel Contour-Buildup Algorithm for the Molecular Surface (2011)		■			■		■				■								
TIALA – Time Series Alignment Analysis (2011)				■	■						■			■	■				■
The Galaxy Track Browser: Transforming the Genome Browser from Visualization Tool to Analysis Tool (2011)				■		■					■		■		■			■	■
Modeling and Visualization of Receptor Clustering on the Cellular Membrane (2011)		■			■		■				■					■			
TVNViewer: An interactive visualization tool for exploring networks that change over time or space (2011)		■			■			■			■				■				
GenAMap: Visualization Strategies for Structured Association Mapping (2011)	■					■			■		■	■	■	■	■	■			■
Visualizing Virus Population Variability From Next Generation Sequencing Data (2011)				■		■					■		■	■	■				■
HiTSEE: A Visualization Tool for Hit Selection and Analysis in High-Throughput Screening Experiments (2011)	■								■		■	■		■					■
Sequence Surveyor: Leveraging Overview for Scalable Genomic Alignment Visualization (2011)				■				■			■				■	■			
Uncertainty-Aware Visual Analysis of Biochemical Reaction Networks (2012)		■								■	■		■	■	■				
ADVISe: Visualizing the dynamics of enzyme annotations in UniProt/Swiss-Prot (2012)	■		■			■		■			■		■	■			■		
Gene-RiViT: A visualization tool for comparative analysis of gene neighborhoods in prokaryotes (2012)				■				■			■			■	■				
Implicit Surfaces for Interactive Graph Based Cavity Analysis of Molecular Simulations (2012)		■			■		■				■		■	■	■				
enRoute: Dynamic Path Extraction from Biological Pathway Maps for In-Depth Experimental Data Analysis (2012)	■	■									■	■	■	■	■				
Heterogeneity-based Guidance for Exploring Multiscale Data in Systems Biology (2012)		■			■						■								■
Dynamic Channels in Biomolecular Systems: Path Analysis and Visualization (2012)		■					■				■			■					
Hive plots — rational approach to visualizing networks (2012)		■				■		■	■		■				■				
Guiding the interactive exploration of metabolic pathway interconnections (2012)		■						■			■		■	■					■
Towards Real-Time Visualization of Detailed Neural Tissue Models: View Frustum Culling for Parallel Rendering (2012)		■			■						■								
Compressed Adjacency Matrices: Untangling Gene Regulatory Networks (2012)		■						■			■		■	■		■			
MaTSE: The Microarray Time-Series Explorer (2012)				■	■						■		■		■	■			
HumMod Browser: An Exploratory Visualization Tool for the Analysis of Whole-Body Physiology Simulation Data (2013)	■				■			■			■		■	■	■				■
STAR: an integrated solution to management and visualization of sequencing data (2013)				■		■		■			■	■	■		■				
COMBat: Visualizing co-occurrence of annotation terms (2013)	■		■			■		■			■		■	■	■		■		
neuroMap – Interactive Graph-Visualization of the Fruit Fly's Neural Circuit (2013)	■						■				■		■	■					
MoClo Planner: Interactive Visualization for Modular Cloning Bio-Design (2013)	■			■				■			■		■	■					
The Molecular Control Toolkit: Controlling 3D Molecular Graphics via Gesture and Voice (2013)		■					■				■			■					
Leveraging Wall-sized High-Resolution Displays for Comparative Genomics Analyses of Copy Number Variation (2013)				■				■			■			■	■				
VisNEST – Interactive Analysis of Neural Activity Data (2013)	■						■				■			■	■				
PresentaBALL – a Powerful Package for Presentations and Lessons in Structural Biology (2013)		■					■				■		■	■					

Technique																			
Large-scale multiple sequence alignment visualization through gradient vector flow analysis (2013)				■		■		■			■				■				
Entourage: Visualizing Relationships between Biological Pathways using Contextual Subsets (2013)	■	■						■	■		■	■		■	■				
Visual Cleaning of Genotype Data (2013)	■			■				■			■		■	■					■
Exploring biological data: Mappings between ontology- and cluster-based representations (2013)		■	■			■		■			■		■	■					
LineUp: Visual Analysis of Multi-Attribute Rankings (2013)	■					■		■	■	■	■	■	■	■	■	■			
Variant View: Visualizing Sequence Variants in their Gene Context (2013)				■				■			■			■	■				
Genome-Wide Detection of sRNA Targets with rNAV (2013)				■				■			■	■	■	■	■				
From Biochemical Reaction Networks to 3D Dynamics in the Cell: the ZigCell3D Modeling, Simulation and Visualisation Framework (2013)		■			■		■				■		■	■	■	■			
Analyzing Chromatin Using Tiled Binned Scatterplot Matrices (2014)	■			■				■	■		■				■				
Genotet: An Interactive Web-based Visual Exploration Framework to Support Validation of Gene Regulatory Networks (2014)	■	■						■	■		■			■		■			
Visualization of gene expression information within the context of the mouse anatomy (2014)	■		■	■	■	■		■			■	■		■	■				
Helium: Visualization of Large Scale Plant Pedigrees (2014)		■	■	■		■	■	■	■		■	■	■	■				■	
Addressing the unmet need for visualizing conditional random fields in biological data (2014)		■		■		■		■	■		■		■	■	■				
ConTour: Data-Driven Exploration of Multi-Relational Datasets for Drug Discovery (2014)	■	■							■		■		■		■				
UpSet: Visualization of Intersecting Sets (2014)	■							■	■		■	■	■	■		■			
inPHAP: Interactive visualization of genotype and phased haplotype data (2014)				■		■		■			■	■	■	■	■			■	■
ReadXplorer – visualization and analysis of mapped sequences (2014)				■		■			■		■	■	■		■				
Domino: Extracting, Comparing, and Manipulating Subsets across Multiple Tabular Datasets (2014)	■							■	■		■	■	■	■		■			
BiNA: a visual analytics tool for biological network data (2014)	■	■			■			■	■	■	■		■	■	■				
Rebuilding KEGG Maps: Algorithms and Benefits (2014)		■					■				■								
MCA: Multiresolution Correlation Analysis, a graphical tool for subpopulation identification in single-cell gene expression data (2014)				■				■	■		■				■				
MIMTool: A Tool for Drawing Molecular Interaction Maps (2014)		■					■	■			■			■				■	
eXamine: Exploring annotated modules in networks (2014)		■	■	■			■	■			■		■	■			■		
iGPSe: A visual analytic system for integrative genomic based cancer patient stratification (2014)	■			■	■	■		■	■		■	■	■	■	■	■			■
VisRseq: R-based visual framework for analysis of sequencing data (2015)	■			■		■		■	■		■		■	■		■			
GOplot: an R package for visually combining expression data with functional analysis (2015)	■		■			■		■	■		■		■						
Visual analysis of biological data-knowledge networks (2015)		■						■	■		■		■	■	■		■		
Integrated visual analysis of protein structures, sequences, and feature data (2015)		■		■		■	■	■			■	■		■	■	■		■	
VisExpress: Visual exploration of differential gene expression data (2015)	■		■	■		■		■	■		■	■	■	■	■	■			
Visual parameter optimisation for biomedical image processing (2015)	■	■						■	■		■		■	■	■		■	■	
Extended LineSets: A visualization technique for the interactive inspection of biological pathways (2015)		■	■				■	■			■	■	■	■	■				■
VISIONET: intuitive visualisation of overlapping transcription factor networks, with applications in cardiogenic gene discovery (2015)		■						■	■		■		■	■					
coMET: visualisation of regional epigenome-wide association scan results and DNA co-methylation patterns (2015)	■							■	■		■			■	■		■		
cnvCurator: an interactive visualization and editing tool for somatic copy number variations (2015)				■		■		■			■			■					
XCluSim: A visual analytics tool for interactively comparing multiple clustering results of bioinformatics data (2015)	■							■	■		■		■	■	■	■			
miRTarVis: An interactive visual analysis tool for microRNA-mRNA expression profile data (2015)	■	■				■		■	■		■	■	■	■				■	
Pan-Tetris: an interactive visualisation for Pan-genomes (2015)	■			■		■		■			■			■				■	
GRAPHIE: graph based histology image explorer (2015)		■						■	■		■	■	■	■	■	■	■	■	■
ReactionFlow: Visualizing Relationships between Proteins and Complexes in Biological Pathways (2015)		■					■	■			■	■	■	■		■			
PathwayMatrix: Visualizing binary relationships between proteins in biological pathways (2015)		■					■	■			■		■	■		■			
LayerCake: a tool for the visual comparison of viral deep sequencing data (2015)				■		■				■	■		■	■	■		■		
Epiviz: a view inside the design of an integrated visual analysis software for genomics (2015)				■				■			■		■	■	■	■		■	
BactoGeNIE: a large-scale comparative genome visualization for big displays (2015)				■		■		■			■		■	■	■	■			
J-Circos: an interactive Circos plotter (2015)				■		■		■	■		■			■	■				
Treelink: data integration, clustering and visualization of phylogenetic trees (2015)				■			■	■			■	■		■	■	■	■		
Vials: Visualizing Alternative Splicing of Genes (2016)		■		■		■		■	■		■			■	■				
CompNet: a GUI based tool for comparison of multiple biological interaction networks (2016)		■						■			■			■	■	■			
HilbertCurve: an R/Bioconductor package for high-resolution visualization of genomic data (2016)				■		■			■		■				■				
Integrated Genome Browser: visual analytics platform for genomics (2016)				■		■					■	■		■	■		■	■	
